# Diseases of the Nose and Throat

**Published:** 1890-01

**Authors:** 


WOOD'S SPECIALTIES IN THE PRACTICE OF MEDICINE—DISEASES OF THE NOSE AND THROAT, by Francke Huntington Bosworth, A. M., M. D., Professor of diseases of the throat in Bellevue Hospital Medical College ; physician to the out-door department of the Bellevue Hospital ; Fellow of the American Laryngological Society of the American Climatological Association of the New York Academy of Medicine, etc.

The first volume of this work has just been placed in our hands, and it is with great pleasure that we review it, and try to give a few of its many good qualities.

While the doctor's first work on the subject was very good, this is so much superior that there is no comparison.

This volume treats simply of the nose and naso-pharynx.

It has been said that the eye is the best teacher. Certainly in this work it will find full employment, for this volume alone contains one hundred and eighty-two wood-cuts and four colored plates. The chapters on the anatomy and physiology of the nose are excellent.

The author has also introduced much matter, hardly touched by others in their works, such as nasal hydrorrhœa and diseases of the accessory sinuses.

A whole section is devoted to the external surgery of the nose, showing by means of wood-cuts both the skin and bone incisions, thus giving in detail all the operations on this organ.

The book is one that should be in every medical man's library, be he specialist or general practitioner. F. O. S.

